# Molecular Profile of a Pituitary Rhabdomyosarcoma Arising From a Pituitary Macroadenoma: A Case Report

**DOI:** 10.3389/fendo.2021.752361

**Published:** 2021-09-29

**Authors:** Jinci Lu, Liam Chen

**Affiliations:** Department of Laboratory Medicine and Pathology, University of Minnesota Medical School, Minneapolis, MN, United States

**Keywords:** pituitary adenoma, rhabdomyosarcoma, TP53, ATRX, LZTR1, NF1

## Abstract

Pituitary sarcoma arising in association with pituitary adenoma is an uncommon finding. Most cases of secondary sarcoma have been noted to arise with a median interval of 10.5 years post radiation. In this case report, we describe a 77-year-old man with an incidental discovery of a pituitary macroadenoma on magnetic resonance imaging (MRI) and underwent radiotherapy. Three years after radiation treatment, there was an acute change in clinical symptoms and increase in tumor size and mass effect on the optic chiasm which prompted surgical resection. A pituitary adenoma along with a separate spindle-cell sarcomatous component was identified in histology. Immunohistochemical stain for muscle markers confirmed a development of pituitary rhabdomyosarcoma (RMS). Molecular profiling of the tumor identified mutations in TP53, ATRX, LZTR1, and NF1. Despite its rarity, characterization of pituitary RMS with immunohistochemistry and molecular studies may provide an insight to its pathophysiological relationship with pituitary adenoma.

## Introduction

RMS is a malignant skeletal muscle sarcoma that commonly occurs in children and rarely in adults. It is uncommon for RMS to arise intracranially, especially within the sellar region. Sellar RMS have been mostly reported in association with radiation for pituitary tumors (1, 2). Though rare, there are a few reported cases of pituitary RMS arising from pituitary adenoma without any prior therapy (3–6). It has been suggested in the literature that the median latency period between radiotherapy and tumor occurrence is 10.5 years (7). In this case report, we present the first molecular characterization of a pituitary RMS arising from a pituitary macroadenoma, three years post radiotherapy.

## Case Report

**Figure 1:**
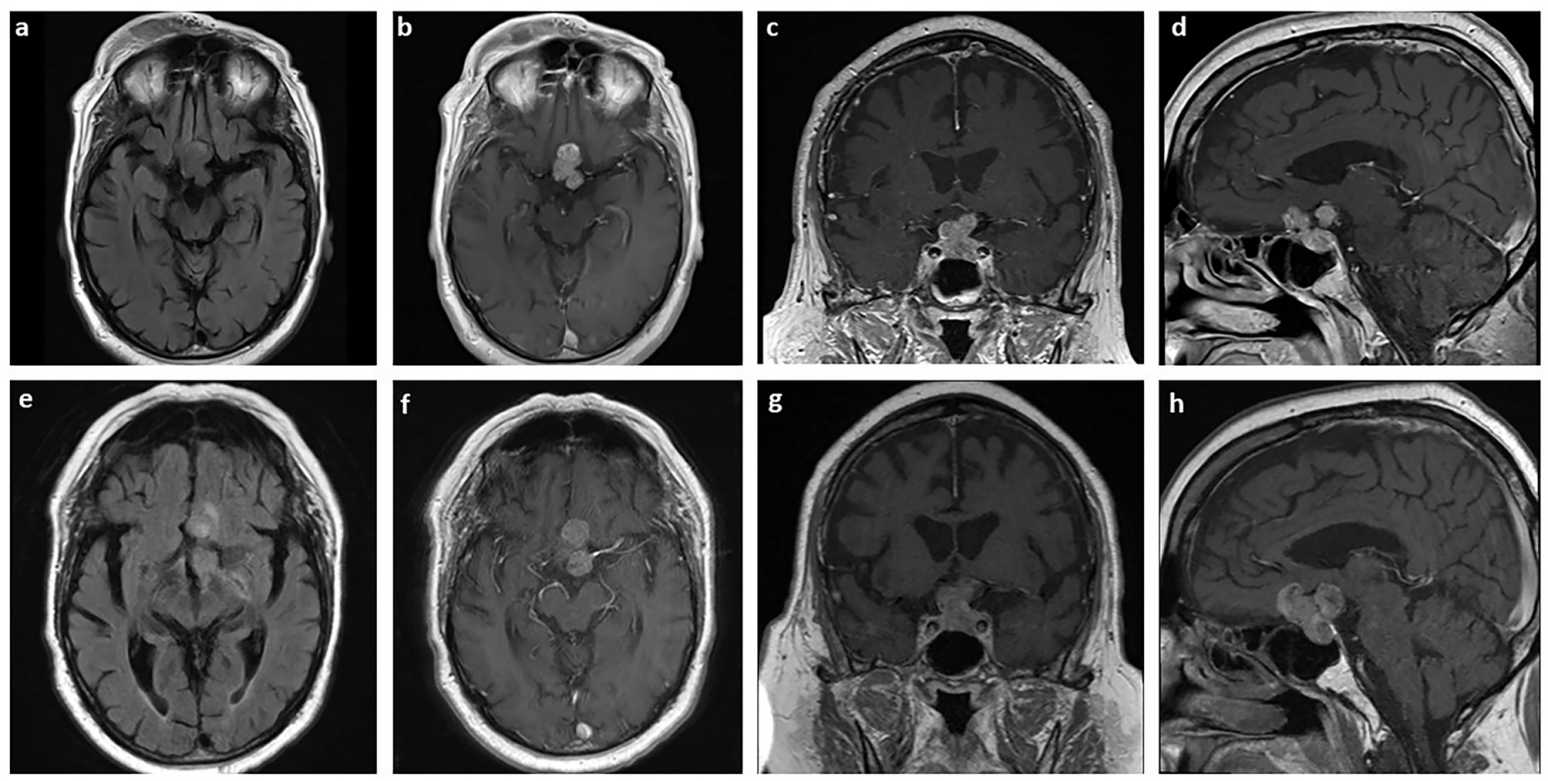
MRI of the pituitary sarcoma arising from a pituitary adenoma. Axial T2 FLAIR **(A)** and axial **(B)**, coronal **(C)**, and sagittal **(D)** T1-weighted with contrast MRI of tumor measuring 2 x 2.7 x 2 cm^3^ at the time of diagnosis. The same sections are shown for the tumor three years later, prior to surgical resection, measuring 3.3 x 3.0 x 2.6 cm^3^ **(E–H)**.

A 77-year-old man with a history of Waldenstrom macroglobulinemia and prostate cancer initially presented to the emergency department complaining of fall. In MRI, an incidental finding of a heterogeneously enhancing and T2 signaling sellar mass with extension to suprasellar cistern measuring 2 x 2.7 x 2 cm^3^ (TR x AP x CC) was identified (**Figures 1A‒D**). Additionally, the lesion encased two-thirds of the left cavernous carotid artery along with significant mass effect on the optic chiasm. The patient was referred to follow up with Neurosurgery and Endocrinology, and it was diagnosed as a pituitary macroadenoma. At the time of diagnosis, the patient had minimal visual field deficits, specifically left-side blurriness. The patient also had secondary hypopituitarism which was replaced with levothyroxine, prednisone, and elected not to take testosterone. Given the patient’s age and comorbidities, it was decided that medical management is the best course of action. The patient underwent 28 fractions of volumetric modulated arc therapy with a total dosage of 5040 cGy.

Over the course of three years, the size of the pituitary mass was relatively stable until early 2021. The mass then was measuring 3.3 x 3.0 x 2.6 cm^3^ with a significant increase in mass effect on adjacent structures (**Figures 1E‒H**). The patient was also experiencing acute change in his vision, especially on their left eye. To preserve the patient’s vision, stealth-guided transnasal endoscopic resection of the pituitary macroadenoma was performed, and specimens were sent for microscopic examination.

**Figure 2:**
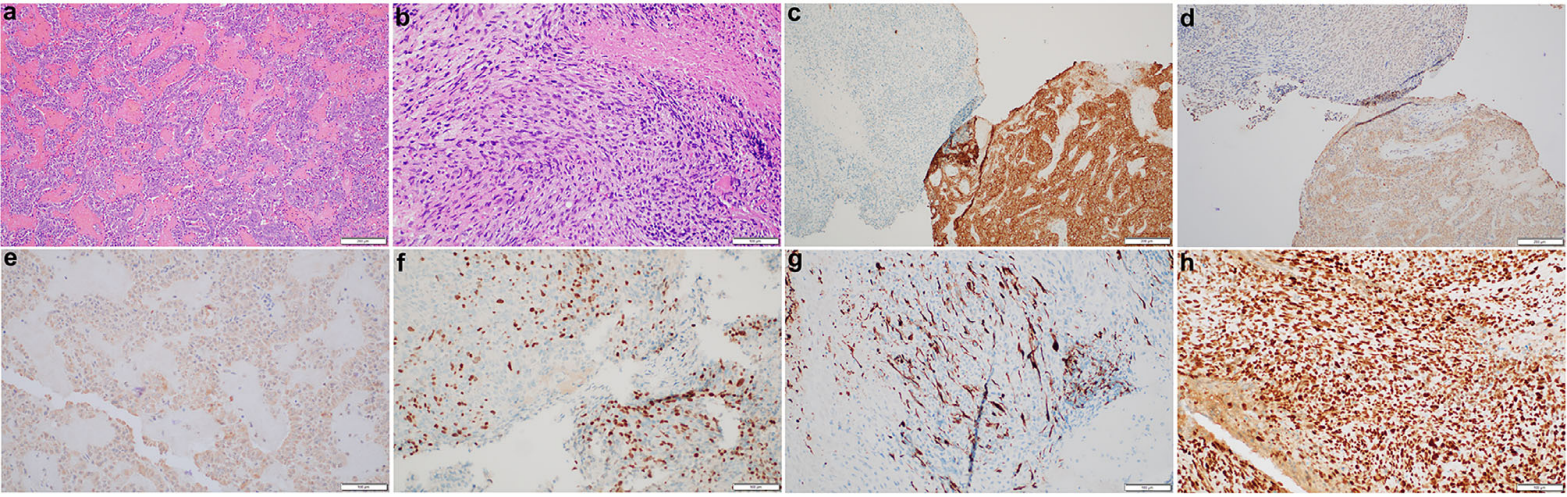
Histopathological and immunohistochemical characterization of the pituitary sarcoma arising from a pituitary adenoma. H&E stains of the pituitary adenoma **(A)** and a concurrent sarcoma which has brisk mitotic activity and necrosis **(B)**. The adenoma is immunoreactive for synaptophysin **(C)**, ACTH **(D)** and GH **(E)** whereas the sarcomatous component has lost synaptophysin stain **(C)**, but become patchy positive for myogenin **(F)**, desmin **(G)**, and diffusely positive for p53 **(H)**.

Histological studies of the resected tumor revealed pituitary adenoma with the loss of acinar architecture and ribbon-like arrays (**Figure 2A**). In addition, there was a presence of spindle-like cells with necrosis and brisk mitotic activity (**Figure 2B**). Immunohistochemical stain for synaptophysin, adrenocorticotropic (ACTH) and growth hormone (GH) was positive for the pituitary adenoma but not the sarcomatous component (**Figures 2C‒E**). Instead, the sarcomatous component was positive for desmin, myogenin, and p53, confirming the diagnosis of a pituitary RMS arising from the pituitary adenoma (**Figures 2F‒H**). NextGen Sequencing (NGS) was also performed on the RMS part, and the following pathological mutations were identified: *TP53* (c.503A>G, p.H168R), *ATRX* (c.5406dup, p.R1803Tfs*7), *LZTR1* (c.791+1G>A), and *NF1* (c.2998del, p.R1000Vfs*12).

## Discussion

Pituitary sarcoma arising from pituitary adenoma are extremely rare, and most cases have been reported to be associated with prior radiation. Although our patient had prior radiation, it was only three years prior compared to the median interval of 10.5 years between radiation and tumor occurrence (7). Thus, it is more likely of a pituitary sarcoma arising from the adenoma rather than a radiation-induced tumor. As proposed by de Silva et al., this may potentially be a metaplastic transformation from the pituitary adenoma or two independent lesions, with the RMS originating from primitive mesenchymal in the dura mater of the sellar floor or in pericapillary space of the pituitary gland (5, 6). Another interesting finding is the presence of multiple genetic mutations in the tumor. *TP53* mutations are common in sarcomas, including leiomyosarcoma, liposarcomas, and RMS (8–12). Loss of *ATRX* is highly associated with alternative lengthening of telomeres, and it is frequently found in complex sarcomas (8, 13–15). Though the mechanism has yet to be elucidated in sarcomas, mutations or deletions of *LZTR1* disrupt RAS regulations by *LZTR1*-mediated ubiquitination and allow glioblastoma to retain its proliferative features (16, 17). Lastly, *NF1* mutations can also be present in sarcomas along with other mutations. Specifically, a study has shown that of 22 primary intracranial spindle cell sarcoma with RMS features, 22% have mutations or deletions of NF1 and 55% have TP53 mutations (11). To our knowledge, this is the first case to report molecular characterizations of a pituitary RMS arising from a pituitary adenoma. Understanding the molecular profile of pituitary sarcoma will help to better understand its etiology and refine treatment plan.

## Data Availability Statement

The original contributions presented in the study are included in the article/supplementary material. Further inquiries can be directed to the corresponding author.

## Ethics Statement

The studies involving human participants were reviewed and approved by University of Minnesota Institutional Review Board. The patients/participants provided their written informed consent to participate in this study.

## Author Contributions

JL collected the history and drafted the manuscript. LC performed the pathological examination and edited the manuscript. All authors contributed to the article and approved the submitted version.

## Conflict of Interest

The authors declare that the research was conducted in the absence of any commercial or financial relationships that could be construed as a potential conflict of interest.

## Publisher’s Note

All claims expressed in this article are solely those of the authors and do not necessarily represent those of their affiliated organizations, or those of the publisher, the editors and the reviewers. Any product that may be evaluated in this article, or claim that may be made by its manufacturer, is not guaranteed or endorsed by the publisher.
